# Multicentric Atrial Strain COmparison between Two Different Modalities: MASCOT HIT Study

**DOI:** 10.3390/diagnostics10110946

**Published:** 2020-11-13

**Authors:** Matteo Cameli, Marcelo Haertel Miglioranza, Julien Magne, Giulia Elena Mandoli, Giovanni Benfari, Roberta Ancona, Gerolamo Sibilio, Vlatka Reskovic Luksic, Dosen Dejan, Leonardo Griseli, Caroline M. Van De Heyning, Philippe Mortelmans, Blazej Michalski, Karolina Kupczynska, Giovanna Di Giannuario, Fiorella Devito, Raluca Dulgheru, Federica Ilardi, Alessandro Salustri, Galal Abushahba, Doralisa Morrone, Iacopo Fabiani, Martin Penicka, Asim Katbeh, Giuseppe Sammarco, Roberta Esposito, Ciro Santoro, Maria Concetta Pastore, Salvatore Comenale Pinto, Artem Kalinin, Žanna Pičkure, Katja Ažman Juvan, Anja Zupan Mežnar, Augustine Coisne, Amandine Coppin, Mihaela Maria Opris, Dan Octavian Nistor, Riitta Paakkanen, Tor Biering-Sørensen, Flemming Javier Olsen, Tomas Lapinskas, Jolanta Justina Vaškelyté, Laura Galian-Gay, Guillem Casas, Andreea Iulia Motoc, Constantinos Hristou Papadopoulos, Savvas Loizos, Gergely Ágoston, Istvan Szabó, Krasimira Hristova, Svetlin Netkov Tsonev, Elena Galli, Dragos Vinereanu, Sorina Mihaila Baldea, Denisa Muraru, Sergio Mondillo, Erwan Donal, Maurizio Galderisi, Bernard Cosyns, Thor Edvardsen, Bogdan A. Popescu

**Affiliations:** 1 Department of Medical Biotechnologies, Division of Cardiology, University of Siena, 53100 Siena, Italy; giulia_elena@hotmail.it (G.E.M.); pastore2411@gmail.com (M.C.P.); mondillo@unisi.it (S.M.); 2 Institute of Cardiology, University Foundation of Cardiology, Porto Alegre 90620-000, Brazil; marcelohaertel@gmail.com (M.H.M.); lgriseli.lg@gmail.com (L.G.); 3 CHU Limoges, Hôpital Dupuytren, Service Cardiologie, F-87042, 87042 Limoges, France; julien.magne@chu-limoges.fr; 4 Cardiology Department, INSERM U1094, Faculté de médecine de Limoges, 2, rue Marcland, 87000 Limoges, France; 5 Section of Cardiology, Department of Medicine, University of Verona, 37126 Verona, Italy; giovanni.benfari@gmail.com; 6 UOC Cardiologia/UTIC—“Santa Maria delle Grazie” Hospital Pozzuoli, 80078 Pozzuoli, Italy; roby.ancona@gmail.com (R.A.); gerolamo.sibilio@aslnapoli2nord.it@gmail.it (G.S.); salvcom@fastwebnet.it (S.C.P.); 7 Department of Cardiovascular Diseases, University Hospital Centre Zagreb, 10000 Zagreb, Croatia; vlatka.reskovic@gmail.com (V.R.L.); dejan.dosen@yahoo.com (D.D.); 8 Department of Cardiology, Antwerp University Hospital, 2650 Edegem, Belgium; caroline.vandeheyning@uza.be (C.M.V.D.H.); philippe.mortelmans@gmail.com (P.M.); 9 Department of Cardiology, Medical University of Lodz, 91347 Lodz, Poland; bwmichalski@gmail.com (B.M.); karolinakupczynska@gmail.com (K.K.); 10 UO Cardiologia, Ospedale Infermi di Rimini, 47923 Rimini, Italy; gdigiannuario@gmail.com; 11 Ramazzini Hospital, 41012 Carpi, Italy; fiorella.devito@alice.it; 12 Cardiology Department—Heart Valve Clinic—University Hospital Liege, B-4000 Liege, Belgium; eralucadulgheru@yahoo.com (R.D.); fedeilardi@gmail.com (F.I.); 13 Department of Advanced Biomedical Sciences, Federico II, University Hospital, 80131 Naples, Italy; robertaesposito.6@gmail.com (R.E.); cirohsantoro@gmail.com (C.S.); mgalderi@unina.it (M.G.); 14 Non-Invasive Department, Heart Hospital—Hamad Medical Corporation, Doha 3050, Qatar; asalustri@hamad.qa (A.S.); gabushahba@hamat.qa (G.A.); 15 Cardiothoracic and Vascular Department, Pisa University, 56126 Pisa, Italy; doralisa.morrone@unipi.it (D.M.); iacopofabiani@gmail.com (I.F.); 16 Cardiologia e Medicina Cardiovascolare—Fondazione Toscana Gabriele Monasterio, 56124 Pisa, Italy; 17 Cardiovascular Research Center Aalst, OLV Clinic, 9300 Aalst, Belgium; martin.penicka@olvz-aalst.be (M.P.); asimkatbeh@yahoo.com (A.K.); 18 Department of Cardiac, Thoracic and Vascular Sciences, University of Padua, 35122 Padua, Italy; giuse.sammarco@libero.it (G.S.); denisa.muraru@gmail.com (D.M.); 19 Department “Gailezers”, Riga East Clinical University Hospital, LV-1038 Riga, Latvia; artem_kalinin@yahoo.com (A.K.); zanna.pickure@gmail.com (Z.P.); 20 Department of Cardiovascular Surgery, University Medical Centre Ljubljana, 1000 Ljubljana, Slovenia; katja.azman@gmail.com; 21 Cardiology Department, University Medical Centre Ljubljana, 1000 Ljubljana, Slovenia; anja.zupanmeznar@gmail.com; 22 Department of Clinical Physiology and Echocardiography—Heart Valve Clinic, Lille University Hospital, 59800 Lille, France; augustine.coisne@chru-lille.fr (A.C.); coppin_amandine@yahoo.fr (A.C.); 23 Institute for Emergency Cardiovascular Diseases and Transplant of Targu Mures, 540136 Targu Mures, Romania; drmihaelaopris@gmail.com (M.M.O.); dr.dan.nistor@gmail.com (D.O.N.); 24 Heart and Lung Center, Helsinki University Hospital and Helsinki University, 00100 Helsinki, Finland; riitta.paakkanen@helsinki.fi; 25 Department of Cardiology, Herlev and Gentofte Hospital, University of Copenhagen, 2900 Copenhagen, Denmark; tor.biering@gmail.com (T.B.-S.); flemming.j.olsen@gmail.com (F.J.O.); 26 Department of Cardiology, Medical Academy, Lithuanian University of Health Sciences, 44307 Kaunas, Lithuania; tomas.lapinskas@lsmuni.it (T.L.); jvaskelyte@gmail.com (J.J.V.); 27 Department of Cardiology, Hospital Universitari Vall d’Hebron, 08035 Barcelona, Spain; lauragaliangay@gmail.com (L.G.-G.); gcasasmasnou@gmail.com (G.C.); 28 Centre for Cardiovascular Diseases, University Hospital of Brussels, B-1090 Brussels, Belgium; andreea.motoc@gmail.com (A.I.M.); bcosyns@gmail.com (B.C.); 29 Korgialenio Benakio—Red Cross Hospital, 115 26 Athens, Greece; papcost@gmail.com (C.H.P.); s.loizos@gmail.com (S.L.); 30 Department of Family Medicine, University of Szeged, H-6725 Szeged, Hungary; agosto.gergely@med.u-szeged.hu (G.A.); sz.istvan.adorjan@gmail.com (I.S.); 31 Department of Noninvasive Functional Diagnostic and Imaging, National Heart Hospital, 1309 Sofia, Bulgaria; khristovabg@yahoo.com (K.H.); svetmed@gmail.com (S.N.T.); 32 Centre Hospitalier Universitaire de Rennes, Inserm, University of Rennes, LTSI—UMR 1099, F-35000 Rennes, France; elena.galli@chu-rennes.fr (E.G.); erwan.donal@chu-rennes.fr (E.D.); 33 Department of Cardiology, University of Medicine and Pharmacy Carol Davila—Emergency and University Hospital, 050474 Bucharest, Romania; vinereanu@gmail.com (D.V.); sorinamihaila1981@gmail.com (S.M.B.); 34 Department of Medicine and Surgery, University of Milano-Bicocca, 20126 Milan, Italy; 35 Center for Cardiological Innovation, Department of Cardiology, Oslo University Hospital, Rikshospitalet, 0372 Oslo, Norway; thor.edvardsen@medisin.uio.no; 36 Faculty of Medicine, University of Oslo, 0315 Oslo, Norway; 37 Department of Cardiology, University of Medicine and Pharmacy “Carol Davila”—Euroecolab, Emergency Institute for Cardiovascular Diseases “Prof. Dr. C. C. Iliescu”, Sos. Fundeni 258, 022328 Bucharest, Romania; bogdan.a.popescu@gmail.com

**Keywords:** speckle tracking echocardiography, left atrial strain, reference point, multi-centric study, standardization

## Abstract

Two methods are currently available for left atrial (LA) strain measurement by speckle tracking echocardiography, with two different reference timings for starting the analysis: QRS (QRS-LASr) and P wave (P-LASr). The aim of MASCOT HIT study was to define which of the two was more reproducible, more feasible, and less time consuming. In 26 expert centers, LA strain was analyzed by two different echocardiographers (young vs senior) in a blinded fashion. The study population included: healthy subjects, patients with arterial hypertension or aortic stenosis (LA pressure overload, group 2) and patients with mitral regurgitation or heart failure (LA volume–pressure overload, group 3). Difference between the inter-correlation coefficient (ICC) by the two echocardiographers using the two techniques, feasibility and analysis time of both methods were analyzed. A total of 938 subjects were included: 309 controls, 333 patients in group 2, and 296 patients in group 3. The ICC was comparable between QRS-LASr (0.93) and P-LASr (0.90). The young echocardiographers calculated QRS-LASr in 90% of cases, the expert ones in 95%. The feasibility of P-LASr was 85% by young echocardiographers and 88% by senior ones. QRS-LASr young median time was 110 s (interquartile range, IR, 78-149) vs senior 110 s (IR 78-155); for P-LASr, 120 s (IR 80-165) and 120 s (IR 90-161), respectively. LA strain was feasible in the majority of patients with similar reproducibility for both methods. QRS complex guaranteed a slightly higher feasibility and a lower time wasting compared to the use of P wave as the reference.

## 1. Introduction

The left atrium (LA) acts as a reservoir receiving blood from the pulmonary veins during ventricular systole and isovolumic relaxation, as a passive conduit during early filling and diastasis, and as a booster pump during late diastole, at atrial contraction [1]. The study of LA function gained attention in recent years, mostly due to deformation imaging and to the growing evidence of prognostic value of the method. Speckle tracking echocardiography (STE), which assesses the LA longitudinal deformation, is the most promising technique for direct evaluation of LA function [2]. It offers opportunities to measure quantitative parameters of LA function but still lacks clear standardization in this setting. There are two methods available for the measurement of LA strain by STE, using different ECG reference points for the analysis: QRS (left atrial strain during reservoir phase, QRS-LASr) and P wave (P-LASr) [3,4]. The recent European Association of CardioVascular Imaging/American Society of Echocardiography (EACVI/ASE) standardization paper [5] on LA imaging using 2D STE, describes both methods and recommends the use of QRS onset as reference point. In particular, the impossibility of the P wave method to be applied in all patients (atrial fibrillation), and the QRS as the zero reference as the easiest tool for the measurement of LA reservoir function, which is the most validated in the literature, are the main indicated reasons in the document to prefer QRS over P wave. A multi-centric study with a head-to-head comparison of LA strain methods in terms of reproducibility, feasibility and time needed for analysis is, however, not currently available. This is the main rational of the Multicentric Atrial Strain COmparison between Two different modalities (MASCOT), the study initiated by the Heart Imagers of Tomorrow (HIT), the young group of the EACVI. MASCOT sought to compare the agreement between 2 operators. The superiority of QRS-LASr over P-LASr was tested as the primary objective. Secondary objectives included: assessment of feasibility and time needed for the analysis with the two modalities; comparison of results between groups; comparison of performance between less experienced and more experienced echocardiographers (young vs. expert).

## 2. Material and Methods

### 2.1. Population of the Study

From 1 July to 31 October 2018, HIT Members and/or Ambassadors were asked to prospectively collect echocardiographic images of three groups of patients referred to echo-laboratories for clinically indicated echocardiograms: healthy subjects, patients with aortic stenosis (AS) and arterial hypertension (AH), included in the LA pressure overload group, and patients with mitral regurgitation (MR) and heart failure (HF), included in LA pressure–volume overload group. Inclusion criteria were: age over 18 years; informed consent. Inclusion criteria for the single groups are described in the Supplementary Material. Exclusion criteria were: valvular prosthesis; permanent or persistent atrial fibrillation (AF); cardiac transplantation; poor acoustic window. Each centre obtained the approval from its own Ethics committee (N° approval Ethics Committee of the coordinating center of Siena, Italy: 12951-2018). All subjects signed an informed consent for inclusion in the study. All procedures were conducted in accordance with the Declaration of Helsinki.

The centres involved in MASCOT HIT study are listed in the Supplementary Material.

### 2.2. Standard Echocardiography

Each echocardiogram was performed by an expert cardiologist using a commercially available system (GE Medical Systems, Northen) equipped with a 1.5–3.6 MHz transducer. All the subjects were studied in the left lateral recumbent position. Standard left ventricular (LV) diameters were measured in long-axis parasternal view. LV and LA volumes were assessed from apical four-chambers and two-chambers views using the biplane modified Simpson’s method, according to current ASE/EACVI recommendations [6]. Maximal and minimal LA volumes were measured at end-systole, just before the mitral (MV) valve opening (at the beginning of the P wave) and at the mitral valve closure, respectively, both in apical four- and two-chambers view. All LA volumes were then indexed to body surface area (BSA). Left ventricular mass was calculated from 2D images and subsequently indexed to BSA. LV diastolic function was assessed according to current recommendations [7]. The E/e′ ratio was calculated as an estimate of LV filling pressures [7]. Measurements of dimensions and longitudinal function of the right ventricle (RV) were made according to the ASE/EACVI recommendations [8]. MV and tricuspid valve assessment and evaluation of valve regurgitation and stenosis severity was assessed according to ESC guidelines [9]. 

### 2.3. Speckle Tracking Echocardiography

A 2D grey-scale apical four- and two-chamber views were acquired, during three consecutive cardiac cycles, with a frame rate of 40–80 fps in each patient. Each exam was performed or verified by a senior imaging expert for quality assurance purposes.

For LA strain analysis, a complete tutorial was provided to each echocardiographer to reduce the risk of bias. The ASE/EACVI document for the standardization of LA deformation imaging by STE was used as a reference [5].

Each Centre analyzed LA strain using off-line semi-automatic 2D strain software (EchoPAC, GE Medical, Milwaukee, WI, USA) by two independent echocardiographers, one young and one senior, blinded to each other.

Young and senior operators were defined according to their echocardiographic experience—that is, <10 and ≥10 years, respectively.

**Figure 1:**
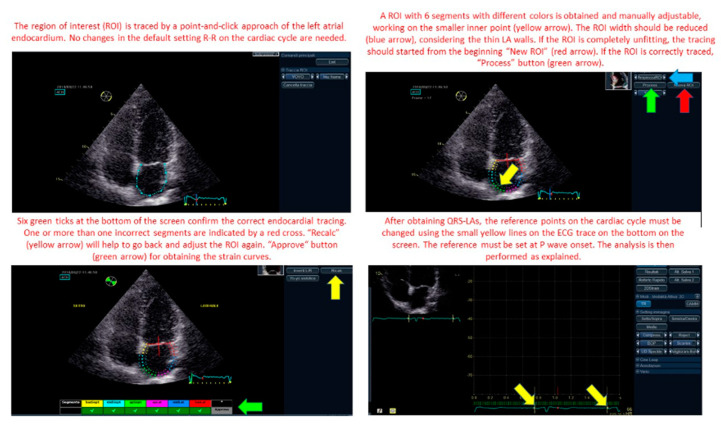
Left atrial (LA_ strain region of interest (ROI) tracing and modification. Zero reference point for LA strain analysis changing from QRS to P wave.

Each echocardiographer calculated LA parameters of longitudinal deformation with both techniques. For QRS method, LA endocardial border was manually traced at LV end-systole in both apical views. The software automatically generated a region of interest (ROI) including six segments with different colours per view. Then, the ROI was manually adjusted to include the thickness of the LA myocardium and optimize tracking quality analysis (Figure 1). A curve was then generated for each of the 12 atrial segments during the QRS-to-QRS cardiac cycle analysis. The ECG reference was then changed on the software to the P wave, leading to a P wave-to-P wave cardiac cycle analysis (Figure 1). The ROI was again traced and adjusted in both apical views and LA strain curves were generated.

The figure shows and explain how to correctly trace the left atrial strain region of interest (ROI) and how to modify the zero reference for the analysis from the QRS to P wave.

The image quality, feasibility and the time needed for LA strain analysis by both methods were also analysed.

LV strain was measured using the QRS complex as a reference time-point and the ROI was manually traced by an endocardial point-and-click approach. The ROI was manually corrected, if needed, in each apical view (four-, two- and three-chambers). The 18-segment model was used and global longitudinal strain (GLS) was reported [10].

### 2.4. Data Collection

The participating centres were chosen among cardiac imaging laboratories with long-time experience in advanced echocardiography and strain analysis, and publications in the field.

Each study investigator was given access to an online platform (REDcap™) with private credentials to MASCOT archive. Data reporting was blinded to the results of the second echocardiographer. Patient data were anonymized, giving a unique code to each patient included in the study, to guarantee privacy accordingly to national and international laws.

### 2.5. Sample Size Justification

See Supplementary Material.

### 2.6. Statistical Analysis

Data are presented as means ± SD, median and interquartile range (for continuous variables), or percentages (for binary variables), as appropriate. Normality was assessed using Kolmogorov-Smirnov test. Comparisons across patient groups were performed using analysis of variance (ANOVA), χ^2^ test, with or without continuity correction. Absolute agreement between young and senior operators was tested using a two-way mixed model considering average measurements. Interrater reliability was tested by Cohen’s Kappa coefficient. Pearson’s correlation coefficients were calculated to assess the relationships between continuous variables in data with normal distribution. ICC was computed by a single-rating, absolute-agreement, and 2-way random-effects model with 3 raters per Centre.

All analyses were performed using SPSS (Statistical Package for the Social Sciences, Version 20.0, SPSS Inc., Chicago, IL, USA). The significance level was set at 0.05 for all analyses.

## 3. Results

### 3.1. General Characteristic of the Enrolling Centres

See Supplementary Material and Table S1.

### 3.2. General Characteristics of the Population

The MASCOT HIT study enrolled 1037 subjects, of which 99 were excluded due to incomplete data provided. The final population was thus composed of 938 subjects: 309 healthy controls; 139 patients with AH, 194 patients with AS (total of 333 in the LA pressure overload condition group); 128 patients with MR and 168 patients with HF (total of 296 included in LA volume-pressure overload group). Mean population age was 59±14 years, 55.7% males. Table 1 describes the clinical characteristics of the study population, while Table 2 presents the standard echocardiographic parameters.

### 3.3. Assessment of Left Atrial Function

**Figure 2:**
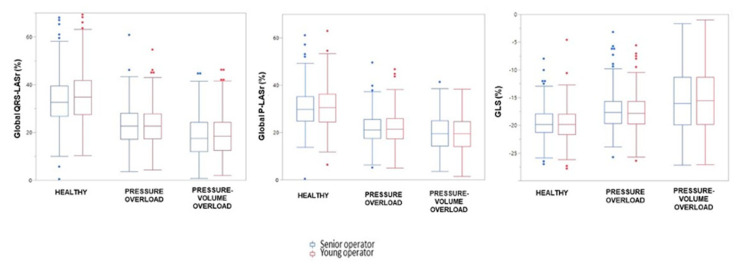
Global QRS-LASr, global P-LASr and GLS in the different study groups. Comparison of strain parameters measurements between senior (blue) and young (red) operators. From the left: global QRS-LASr, global P-LASr and left ventricular GLS. LASr = left atrial strain during reservoir phase; GLS = global longitudinal strain.

The values of global QRS-LASr, QRS-LASct and P-LASr in the different groups are showed in Table 3. Figure 2 presents box and whisker plots for global QRS-LASr, P-LASr and GLS.

The inter-operator reproducibility by ICC was excellent for both measures of LA strain with comparable values: 0.93 for global QRS-LASr and 0.90 for global P-LASr. The reproducibility of LA strain was close to the ICC value for LV GLS (0.96). When analysing the study groups separately, we found that the reproducibility was better in pathological left atria compared to healthy individuals, with the best results in the pressure–volume overload model. All the ICC values are summarized in Table 4.

Variability of QRS-LASr and P-LASr values was not affected by LA volume or E/e’ ratio. Even if a trend of higher reproducibility can be found in MASCOT data both for QRS and P measurements in patients with dilated LA or elevated filling pressures, this did not reach statistical significance.

Young echocardiographers were able to analyze QRS-LASr in both apical views in 90% of subjects, in only one apical view (4 or 2 chambers) in 9%, and the analyses were not obtainable in only 1% of cases. Senior operators reported an overall QRS-LASr feasibility of 95%. Substantial feasibility agreement was found between young and senior echocardiograpgers (Cohen’s Kappa 0.63). These values were similar to those for LA volume and LVEF feasibility (97% and 95%, respectively). The feasibility of P-LASr method in young echocardiographers was 85% in both views, 12% in only one apical view, while 3% of cases were not feasible. The feasibility of the P-LASr method in senior echocardiographers was 88%, with a Kappa coefficient of 0.48. Experience with strain analysis was not associated with the time needed for analysis. QRS-LASr required less time to be obtained than the P-LASr. The median time to perform the measurements for QRS-LASr was 110 s (IR 78-149) and 110 s (IR 78-155) in young and senior echocardiographers, respectively. The median time to measure global P-LASr was 120 s (IR 80-165) and 120 s (IR 90-161) for young and senior echocardiographers, respectively.

Both QRS-LASct and P-LASct methods evaluate the contractile function of the LA. From our data, Pearson’s correlation analysis revealed a good correlation between the two methods, both when assessed by young and by senior operators (Table 5). A strong correlation was also found between the two indices of reservoir function.

## 4. Discussion

The main findings of the MASCOT HIT study are: (1) LA strain analysis provides excellent inter-operator variabilities when using both methods, only slightly lower compared to LV GLS; (2) the measurement of global QRS-LASr is more feasible than global P-LASr with a substantial agreement between the senior and the young echocardiographers; (3) assessment of LA strain by QRS method is quite faster; (4) there is an overall good correlation between the values of QRS-LASr and P-LASr (indices of LA compliance and reservoir function), better in patients with LA pressure and pressure–volume overload than in healthy controls.

The importance of the assessment of LA function over LA size is becoming increasingly appropriate, not only for research purposes but also for everyday practice [11,12,13]. After the application of STE to other chambers than the LV, the additional role of LA deformation imaging has been explored in several clinical settings, mostly including conditions of atrial volume or pressure–volume overload e.g., heart valve diseases [14,15,16,17], AH [18,19], HF [20,21,22,23]. This led to the conclusion that a reduced LA longitudinal strain could be useful for the diagnosis, management and prognostic stratification in several conditions.

Some authors argued that analysing LA phases based on R-wave might differ among patients, due to the fact that R-wave is related to the LV depolarization, and not to the LA [24]. Previous studies demonstrated a close correlation between atrial and ventricular dynamics, underlining the concordance between the mitral annulus motion with LV mechanics during the entire cardiac cycle. Wakami et al. [25] confirmed this hypothesis by finding a significant correlation between peak LA strain and LV systolic longitudinal strain. Moreover, the strong correlation between QRS-LASr and invasively measured LV end-diastolic pressure shows the close interdependence between LA and LV function during the entire cardiac cycle, suggesting QRS-LASr may have a role as marker of atrial-ventricular interplay.

In the three groups of patients evaluated in the MASCOT HIT study, similar reproducibility but superiority of QRS method, in terms of feasibility and time needed for the analysis, emerged from the study’s results. Strain analysis showed a trend to a better reproducibility in patients with higher LA volume; however, statistical significance was not reached. This could be explained by the fact that tracing the endocardial border is generally easier in dilated atria and the software is more capable to follow the displacement of speckles. The reproducibility of QRS-LASr was slightly superior to P-LASr in MASCOT HIT study population and in the three groups separately.

Current analytical software for calculating strain values are customized for R-wave zero-reference point so they automatically generate the frame where the endocardial tracing must be started. On the contrary, additional manipulations are needed to set the onset of P wave as the trigger and this procedure is done on the ECG trace acquired with the echocardiographic image. This aspect leads to important consequences: first, the arbitrariness of choosing the starting frame on the ECG is responsible for a higher operator dependence and for lower inter-operator reproducibility and agreement according to operator experience. Second, the difficulty to obtain a good ECG trace where the P wave can be clearly defined reduces the feasibility of the method. Third, the search for a readable ECG where the operator can work during pre-analysis manipulation extends the time both during the echocardiographic study itself and during the off-line analysis. We decided to exclude patients with persistent or permanent AF but the impossibility to perform LA strain using the P-wave method in this clinical setting is not negligible. These are important aspects from both clinical and research perspectives. The overall reproducibility and feasibility of LA strain can also be limited by an increased heart rate. Tachycardia particularly interferes with a correct P wave identification and with the discrimination between P and T waves in suboptimal ECG tracing.

QRS-LASr and P-LASr demonstrated a good correlation in evaluating LA reservoir function in MASCOT HIT results, higher in patients than in control group. However, using a sum of parameters instead of a single index might decrease measurement accuracy, with possible mathematical errors and longer time needed for the analysis. Moreover, the strain curve measured by the QRS method seems to follow more closely the LA physiology.

### 4.1. Study Limitations

Intra-operator reproducibility for strain parameters in each Centre was not tested. However, MASCOT HIT involved international imaging centres with high experience in advanced echocardiography and STE. The deformation analysis was performed on single-vendor machine. This software was designed for the analysis of LV strain and then applied to the other cardiac chambers without being specifically designed for LA function analysis. However, at present, the application of this software for LA strain measurement is widely used in practice and is the most commonly used one in the published studies. 

### 4.2. Clinical Perspectives

LA strain assessment in different clinical settings has provided clear pathophysiological insights in addition to its diagnostic and prognostic relevance as demonstrated in several studies. However, there are some factors that still limit its wider use, mainly technical issues related to measurement standardization, choice of parameter to use and/or specific values to be used as cut-off in different settings. On the contrary, LV GLS has already overcome some of these practical aspects, being recommended on top of standard echocardiographic parameters in several clinical settings (e.g., early detection of cardiotoxicity in oncologic patients). This study provides relevant practical information about measuring LA strain and may represent a step forward for its better use in clinical practice.

## 5. Conclusions

The increasing clinical use of LA strain as an index of LA function requires proper standardization for its analysis. Both QRS-LASr and P-LASr methods show high reproducibility, feasibility and short time of measurement. QRS-LASr has a greater feasibility and a shorter analysis time, both for senior and for younger echocardiographers. Considering these data and the impossibility to perform P-LASr measurement in AF patients, QRS-LASr should be considered the preferred parameter to use for LA strain analysis.

## Figures and Tables

**Table 1:** Demographic and clinical characteristics.

	Controls (n = 309)	AH and AS (n = 333)	MR and HF (n = 296)	*p* Value
Age, years	47.1 ± 15.6	65.3 ± 12.9	66.2 ± 13.8	<0.0001
Female, %	47.7	47.4	38.4	0.029
Weight, kg	71.9 ± 13.3	77.8 ± 15.8	75 ± 15.2	<0.0001
Height, cm	170 ± 9.6	167.6 ± 9.4	168.4 ± 9.3	0.0013
BMI, kg/m^2^	24.8 ± 3.9	27.7 ± 4.8	26.4 ± 4.7	<0.0001
BSA, m^2^	1.82 ± 0.2	1.86 ± 0.2	1.84 ± 0.2	0.044
HR, bpm	68.9 ± 11.2	68.9 ± 10.5	70.2 ± 13	0.273
SBP, mmHg	123.4 ± 14	135.7 ± 18.4	127.4 ± 20.7	<0.0001
DBP, mmHg	76 ± 8.5	79.6 ± 11.4	76.2 ± 12.7	<0.0001

AH = Arterial hypertension; AS = Aortic stenosis; BMI = Body Mass Index; BSA = Body Surface Area; DBP = Diastolic blood pressure; HF = Heart Failure; HR = heart rate; MR = Mitral Regurgitation; SBP = Systolic blood pressure.

**Table 2:** Standard echocardiographic parameters.

	Controls (n = 309)	AH and AS (n = 333)	MR and HF (n = 296)	*p* Value
IVS, mm	9.0 ± 1.6	12 ± 2.4	10.8 ± 2.2	<0.0001
LV PW, mm	8.6 ± 1.6	10.9 ± 1.9	10.1 ± 2.0	<0.0001
LV mass index, g/m^2^	75.6 ± 19.1	107.6 ± 31.9	120.4 ± 35	<0.0001
LV EDD, mm	47.3 ± 5.7	47.4 ± 6.2	55.0 ± 8.6	<0.0001
LV ESD, mm	30.6 ± 5.7	31.0 ± 6.6	39.8 ± 11.0	<0.0001
LV EDV index, mL/m^2^	51.2 ± 12.5	51.9 ± 14.4	75.2 ± 30.2	<0.0001
LV ESV index, mL/m^2^	20.4 ± 6.5	21.9 ± 9.2	41.7 ± 28.9	<0.0001
LV EF, %	60.4 ± 6.7	58.5 ± 9.2	47.0 ± 14.6	<0.0001
LA max volume index, mL/m^2^	26.0 ± 6.7	35.1 ± 13.4	45.9 ± 19.0	<0.0001
LA preA volume index, mL/m^2^	16.7 ± 5.8	25.1 ± 12.5	33.3 ± 15.7	<0.0001
LA min volume index, mL/m^2^	10.3 ± 4.3	16.3 ± 10.4	24.2 ± 14.4	<0.0001
E/A ratio	1.3 ± 0.5	1.0 ± 0.5	1.48 ± 1.0	<0.0001
Mitral E DT, ms	196.8 ± 53.4	223.5 ± 76.8	199.4 ± 76.7	<0.0001
E/e’ ratio	7.0 ± 2.8	11.8 ± 6.3	13.4 ± 7.9	<0.0001

AH = Arterial hypertension; AS = Aortic stenosis; DT = Deceleration time; EDD = End-diastolic diameter; EDV = End-diastolic volume; EF = Ejection fraction; ESD = End-systolic diameter; ESV = End-systolic volume; HF = Heart Failure; IVS = Interventricular septum; LA = Left atrial; LV = Left ventricular; MR = Mitral Regurgitation; PW = posterior wall.

**Table 3:** Speckle tracking echocardiography parameters.

	Controls (n = 309)	AH and AS (n = 333)	MR and HF (n = 296)	*p* Value
Global QRS-LASr *y*, %	35.4 ± 11.7	22.9 ± 8.4	19.1 ± 8.9	<0.0001
Global QRS-LASr *s*, %	33.5 ± 10.9	23.0 ± 8.5	18.9 ± 9.2	<0.0001
Global QRS-LASct *y*, %	15.5 ± 5.4	13.3 ± 5.5	10.1 ± 5.7	<0.0001
Global QRS-LASct *s*, %	15 ± 5.3	13.4 ± 5.7	10 ± 5.7	<0.0001
Global P-LASr *y*, %	31.2 ± 8.5	21.8 ± 6.9	19.2 ± 7.6	<0.0001
Global P-LASr *s*, %	30.5 ± 8	21.9 ± 6.8	19.2 ± 7.4	<0.0001
LV GLS *y*, %	−19.9 ± 3.1	−17.6 ± 3.3	−15.5 ± 5.7	<0.0001
LV GLS *s*, %	−19.7 ± 3.0	−17.4 ± 3.3	−15.3 ± 5.4	<0.0001

AH = Arterial hypertension; AS = Aortic stenosis; GLS = Global longitudinal strain; HF = Heart Failure; LV = Left Ventricular; MR = Mitral Regurgitation; LASct = Left atrial strain during contraction phase phase with P as starting point; P-LASr = Left atrial strain during reservoir phase with P as starting point; QRS-LASct = Left atrial strain during contraction phase with QRS as starting point; QRS-LASr = Left atrial strain during reservoir phase with QRS as starting point; P; y = young; s = senior.

**Table 4:** Intraclass Correlation Coefficient (ICC).

	ICC	95% CI Lower Bound	95% CI Upper Bound
**Study population**			
Global QRS-LASr			
Average measures	0.93	0.92	0.94
Global P-LASr			
Average measures	0.90	0.89	0.92
GLS			
Average measures	0.96	0.95	0.96
**Controls**			
Global QRS-LASr			
Average measures	0.84	0.80	0.88
Global P-LASr			
Average measures	0.80	0.75	0.85
**LA pressure overload**			
Global QRS-LASr			
Average measures	0.92	0.90	0.94
Global P-LASr			
Average measures	0.90	0.87	0.92
**LA volume-pressure overload**			
Global QRS-LASr			
Average measures	0.95	0.93	0.96
Global P-LASr			
Average measures	0.94	0.92	0.95

GLS, global longitudinal strain; P-LASr = Left atrial strain during reservoir phase with P as starting point; QRS-LASr = Left atrial strain during reservoir phase with QRS as starting point.

**Table 5:** Values of r coefficients for the correlation between indices of LA reservoir function (QRS-LASr and P-LASr) and of LA contraction (QRS-LASct and P-LASct).

	P-LASr	P-LASct	*p*
CONTROLS measured by young operators			
QRS-LASr	0.76		<0.001
QRS-LASct		−0.47	<0.001
CONTROLS measured by senior operators			
QRS-LASr	0.71		<0.001
QRS-LASct		−0.52	<0.001
LA PRESSURE OVERLOAD measured by young operators			
QRS-LASr	0.82		<0.001
QRS-LASct		−0.71	<0.001
LA PRESSURE OVERLOAD measured by senior operators			
QRS-LASr	0.80		<0.001
QRS-LASct		−0.67	<0.001
LA PRESSURE–VOLUME OVERLOAD measured by young operators			
QRS-LASr	0.87		<0.001
QRS-LASct		−0.69	<0.001
LA PRESSURE–VOLUME OVERLOAD measured by senior operators			
QRS-LASr	0.88		<0.001
QRS-LASct		−0.82	<0.001

LA, left atrial; P-LASct = Left atrial strain during contraction phase phase with P as starting point; P-LASr = Left atrial strain during reservoir phase with P as starting point; QRS-LASr = Left atrial strain during reservoir phase with QRS as starting point; QRS-LASct = Left atrial strain during contraction phase with QRS as starting point.

## Supplementary Materials

The following are available online at https://www.mdpi.com/2075-4418/10/11/946/s1.
