# Supplementary material for: Multicentric Atrial Strain COmparison between Two Different Modalities: MASCOT HIT Study
Source: Diagnostics (Basel). 2020 Nov 13;10(11):946. doi: 10.3390/diagnostics10110946 (PMC7696899; doi:10.3390/diagnostics10110946)
Supplement: Supplementary file 1 [file diagnostics-10-00946-s001.pdf]

Supplementary Data

Groups including criteria

Criteria to be inserted in the AH group included: systolic blood pressure ≥ 140 mmHg and/or diastolic blood pressure ≥ 90 mmHg on three or more occasions or as antihypertensive treatment in previously documented history of AH, according to European Society of Hypertension and European Society of Cardiology (ESC) guidelines (6). Patients with at least mild AS (mean aortic gradient >20 mmHg and/or aortic valve area <1.5 cmq) were included in AS group; patients with MR were included in the corresponding group when presenting a vena contracta >3 mm, regurgitant fraction >30%, EROA >0.2 cmq. HF group included patients with typical sign and symptoms with an ejection fraction (EF) <50% or preserved EF with evidence of diastolic dysfunction.

Sample size justification

According to previous literature, P-LASr and QRS-LASr may have respectively ICC=0.85 and 0.95. To limit risk of a lower difference in our study related to its specific design, we decided to use an expected difference between the ICC of the 2 parameters of 5%. In this regard, using α=0.05, β=0.1 and a risk of drop-out or missing value of 15%, the required sample size to appropriately address the primary objective of the study would be n=80. We deliberately decided to apply this sample size to each studied group (N=3) and to increase it up to n=120 to comply with all secondary objectives of the study. Consequently, a total of 360 patients was required for the present study.

List of MASCOT HIT Centres

The MASCOT HIT study involved 26 imaging Centres around the world:

1. Department of Medical Biotechnologies, Division of Cardiology, University of Siena (Siena, Italy)
2. Institute of Cardiology / University Foundation of Cardiology (Porto Alegre, Brazil)".
3. UOC Cardiologia/UTIC - "Santa Maria della Grazie" Hospital Pozzuoli (Pozzuoli, Italy)
4. Department of Cardiovascular Diseases, University Hospital Centre Zagreb (Zagreb, Croatia)
5. Department of Cardiology, Antwerp University Hospital (Edegem, Belgium)
6. Department of Cardiology, Medical University of Lodz (Lodz, Poland)
7. UO Cardiologia Ospedale Infermi di Rimini (Rimini, Italy) / Ramazzini Hospital, Carpi (Carpi, Italy)
8. Cardiology Department - Heart Valve Clinic - University Hospital Liege (Liege, Belgium)
9. Non-Invasive Department, Heart Hospital - Hamad Medical Corporation (Doha, Qatar)
10. Cardiothoracic and Vascular Department, Pisa University (Pisa, Italy)
11. Cardiovascular Research Center Aalst, OLV Clinic (Aalst, Belgium)
12. Department of Cardiac, Thoracic and Vascular Sciences, University of Padua (Padua, Italy)
13. Department of Advanced Biomedical Sciences, Federico II, University Hospital (Naples, Italy)
14. Riga East Clinical University hospital, Department "Gailezers" (Riga, Latvia)
15. University Medical Centre Ljubljana (Ljubljana, Slovenia)
16. Department of Clinical Physiology and Echocardiography – Heart Valve Clinic, Lille University Hospital (Lille, France)

17. Institute for Emergency Cardiovascular Diseases and Transplant of Targu Mures (Targu Mures, Romania)
18. Department of Cardiology, Herlev and Gentofte Hospital, University of Copenhagen (Copenhagen, Denmark)
19. Department of Cardiology, Medical Academy, Lithuanian University of Health Sciences (Kaunas, Lithuania)
20. Department of Cardiology, Hospital Universitari Vall d'Hebron (Barcelona, Spain)
21. Centre for Cardiovascular Diseases/University Hospital of Brussels (Brussels, Belgium)
22. Korgialenio Benakio - Red Cross Hospital (Athens, Greece)
23. University of Szeged, Department of Family Medicine (Szeged, Hungary)
24. University National Heart Hospital, Sofia (Sofia, Bulgaria)
25. University of Rennes, CHU Rennes - Centre Hospitalier Universitaire de Rennes (Rennes, France)
26. University of Medicine and Pharmacy Carol Davila - Emergency and University Hospital (Bucharest, Romania)

General characteristic of the enrolling centres

Table S1 shows the most active Centres with the number of recruited patients.

Siena was the coordinating Centre. The Centres with the highest number of enrolled patients were Siena (Italy), Pozzuoli (Italy) and Zagreb (Croatia). Among the participating centres, only six had not an echocardiographic accreditation, while the majority had a national one; all the centres perform advance echocardiography, including 3D modality. Regarding young operators, the majority performed between 300 and 1000 echocardiographic exams per year, with an experience of 3-8 years; most of the senior Supervisors did 1000-2000 exams/year with an experience in the field of more than 8 years (mainly between 10 and 20 years).

Table S1. Centers with the highest number of enrolled patients

Centre	Number of enrolled patients
**Department of Medical Biotechnologies, Division of Cardiology, University of Siena (Siena, Italy)**	100
**UOC Cardiologia/UTIC "Santa Maria della Grazie" Hospital Pozzuoli (Pozzuoli, Italy)**	67
**University Hospital Centre Zagreb (Zagreb, Croatia)**	57
**Institute of Cardiology / University Foundation of Cardiology (Porto Alegre, Brazil)**	50
**Antwerp University Hospital (Edegem, Belgium)**	45
**Department of Cardiology, Medical University of Lodz (Lodz, Poland)**	43
**UO Cardiologia Ospedale Infermi di Rimini (Rimini, Italy) / Ramazzini Hospital, Carpi (Carpi, Italy)**	41
**Cardiology Department - Heart Valve Clinic - University Hospital Liege (Liege, Belgium)**	37
